# Using Proteomics to Understand How *Leishmania* Parasites Survive inside the Host and Establish Infection

**DOI:** 10.3390/ijms17081270

**Published:** 2016-08-19

**Authors:** Patrícia Sampaio Tavares Veras, Juliana Perrone Bezerra de Menezes

**Affiliations:** 1 Laboratório de Patologia e Biointervenção, Instituto Gonçalo Moniz, FIOCRUZ, Salvador 40296-710, Brazil; juliana.menezes@bahia.fiocruz.br; 2 Instituto Nacional de Ciência e Tecnologia para Doenças Tropicais (INCT-DT), Salvador 40110-160, Brazil

**Keywords:** proteomics, *Leishmania*, intracellular survival

## Abstract

*Leishmania* is a protozoan parasite that causes a wide range of different clinical manifestations in mammalian hosts. It is a major public health risk on different continents and represents one of the most important neglected diseases. Due to the high toxicity of the drugs currently used, and in the light of increasing drug resistance, there is a critical need to develop new drugs and vaccines to control *Leishmania* infection. Over the past few years, proteomics has become an important tool to understand the underlying biology of *Leishmania* parasites and host interaction. The large-scale study of proteins, both in parasites and within the host in response to infection, can accelerate the discovery of new therapeutic targets. By studying the proteomes of host cells and tissues infected with *Leishmania*, as well as changes in protein profiles among promastigotes and amastigotes, scientists hope to better understand the biology involved in the parasite survival and the host-parasite interaction. This review demonstrates the feasibility of proteomics as an approach to identify new proteins involved in *Leishmania* differentiation and intracellular survival.

## 1. Introduction

*Leishmania* is a protozoan parasite that causes a broad range of different clinical symptoms in humans known as leishmaniasis. This disease represents a major public health problem and is endemic in 98 countries across five continents, Asia, Africa, Europe, North America and South America. Over 350 million people are at risk, with an estimated 12 million infected, and 0.9–1.6 million new cases emerging per year. More than 90% of global visceral leishmaniasis (VL) cases occur in six countries: Bangladesh, Brazil, Ethiopia, India, South Sudan and Sudan. In addition, ten countries with the highest estimated case counts for the cutaneous form of the disease are: Afghanistan, Algeria, Brazil, Colombia, Costa Rica, Ethiopia, Iran, Peru, Sudan, and Syria, together accounting for 70% to 75% of the global estimated cutaneous leishmaniasis (CL) incidence [1,2].

Leishmaniasis is caused by different species of protozoan parasites belonging to the genus *Leishmania*. Different species of *Leishmania* are responsible for varying clinical forms of leishmaniasis. Human leishmaniasis consists of a range of diseases which can manifest as a simple self-limiting or asymptomatic CL to a disfiguring and debilitating VL, the clinical form of the disease associated with higher mortality. Post-kala-azar dermal leishmaniasis (PKDL) is a dermal complication of VL and is considered a reservoir for *Leishmania* parasites [3]. *Leishmania* (*L.*) *major* or *L. tropica* causes localized cutaneous lesions that are usually self-healing [4]. South American species, such as *L. braziliensis*, manifest initially as cutaneous lesions that may metastasize resulting in mucocutaneous lesions or diffuse CL. On the other hand, infections caused by *L.* *donovani* or *L.* *infantum* may lead to chronic disseminating diseases, mainly in the liver and spleen, which are often fatal if left untreated [4].

The advances in large-scale technologies, such as proteomics, have allowed the identification and characterization of pathways, both in the parasite and the host, which have proven to be more effective than studying individual molecules. Proteomics is the large-scale characterization of the proteins in a cell line, tissue, or organism, with the goal to access a more global and integrated view of the biological processes by studying all the proteins in a cell rather than each one individually [5]. The use of proteomics tools has revolutionized several biomedical fields such as medicine and dentistry. Proteomics has contributed greatly to the dentistry field by helping in the identification of different biomarkers present in the oral fluids for early diagnosis of several diseases [6]. Also, proteomics has contributed to the understanding and identification of several medically important biomarkers for different diseases [7,8,9,10]. In the last decade, high-throughput techniques, which can process and analyze large amounts of diverse molecules using automated systems, has enabled us to identify molecules involved in the establishment of diseases caused by *Leishmania* parasites, development of parasite resistance [11,12,13], as well as the characterization of new chemotherapeutic targets [14,15]. The relatively weak correlation between mRNA and protein levels led to the conclusion that it is not possible to predict protein expression based on quantitative mRNA data [16,17]. The above reinforces the idea that proteomics should be considered as a large-scale critical tool to understand the host-*Leishmania* interactions better. Indeed, proteomic studies have been widely used to characterize molecules and pathways expressed in the parasite, as well as in the invertebrate [18,19,20,21], or mammalian [22] hosts.

**Figure 1:**
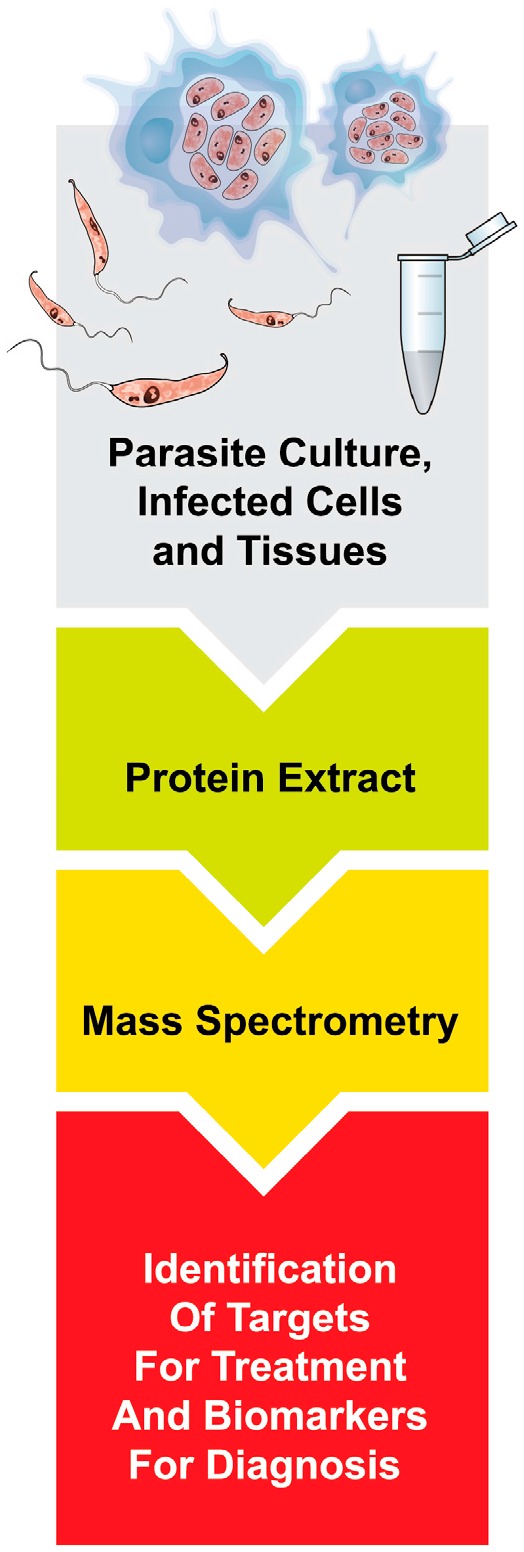
Proteomics approach process. The study of the proteome of *Leishmania*-infected cells and tissues, using mass spectrometry, can lead to the identification of targets for prophylactic and chemotherapeutic treatment and to the identification of biomarkers that can be used for the diagnosis of different diseases. The aim of the present report is to review the recent contributions of proteomics to the understanding of the various aspects of the *Leishmania*-mammalian host interaction. We will first describe the contributions made by large-scale proteomic studies on alterations of protein expression in parasites during their differentiation process from promastigotes to amastigotes, followed by studies identifying proteins differentially expressed by host cells and tissues in response to infection. These recent studies explored proteins expressed in macrophage-*Leishmania* interaction in vitro [22,23], in cutaneous lesions of infected humans [26], as well as in serum of individuals with VL [27,28,29].

In the *Leishmania* research field, proteomic studies have provided valuable insights into the identification of molecules and pathways involved in host-parasite interactions in the parasite [18,19,20,21], and in the host [22,23]. Also, proteomics has significantly contributed to the identification of targets for prophylactic or chemotherapeutic treatment [22,24], as well as biomarkers that can be used for the diagnosis of the different diseases [25] (Figure 1).

## 2. Leishmania Adaptation to the Intracellular Life-Cycle: Modulation in Parasite Protein Expression

### 2.1. Modulation of Proteins during Axenic Differentiation of Leishmania Parasites

**Figure 2:**
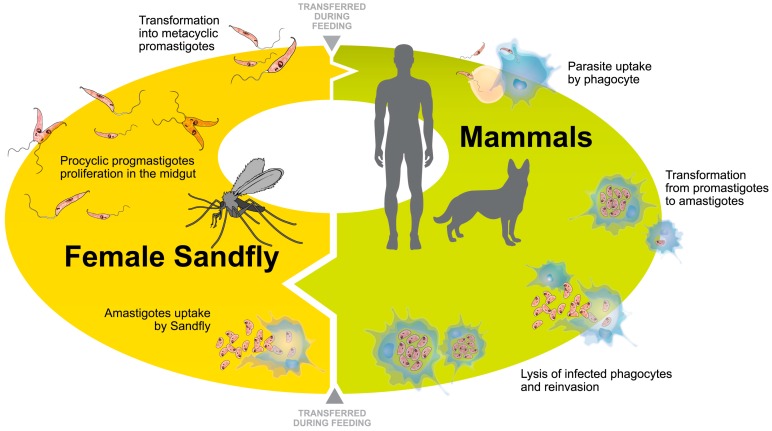
The life cycle of *Leishmania* parasites. During blood feeding by female sandflies, metacyclic promastigotes are regurgitated. These promastigotes are then phagocytosed by cells at the site of the bite. Once inside the host cells, metacyclic promastigotes transform into amastigotes, which can survive and replicate inside phagolysosomes. Amastigote replication may lead to host cell rupture, allowing reinfection of other phagocytes. When infected phagocytes are taken up by another sandfly during the blood meal, amastigotes transform into procyclic promastigotes in the sandfly midgut. *Leishmania* procyclic promastigotes then differentiate into infective metacyclic promastigotes, completing the cycle.

During their life cycle, *Leishmania* spp. adapt to different environments in the insect and the mammalian host by undergoing a variety of morphological and biochemical changes [30,31,32]. These changes in environment correlate with the process of differentiation from promastigote, the motile form that proliferates inside the alimentary tract of Phlebotomine sandflies, to the amastigote form, the non-motile form that multiplies inside the acidified phagolysosomes of mammalian host macrophages [19,33,34,35,36] (Figure 2). The adaptation of the parasite to the host environment is crucial to the differentiation process. This adaptation includes changes in temperature and pH [32], as well as adjustments to the cytotoxic environment of the host. Furthermore, this adaptation is essential for the intracellular survival of the parasite, which requires a combination of survival factors expressed by parasites at distinct stages [37,38]. Until now, the cellular and molecular mechanisms involved in the differentiation of *Leishmania* parasites were poorly understood [19].

The proteomic studies published so far have been conducted using either *L. donovani* or *L. infantum*, probably due to the medical importance of the visceral forms arising from these parasites, which result in high mortality rate. The first study that compared the differentially expressed proteins of *L. infantum* parasites in differing life cycle stages employed comparative two-dimensional gel electrophoresis (2-DE) in addition to mass spectrometry. In this report, more than 62 differentially expressed proteins were detected in axenic amastigotes among ~2000 protein spots resolved by 2-DE. Two-dimensional gel electrophoresis has frequently been used as a protein separation method before mass spectrometry. Spots represent one or more proteins that have migrated to a particular location on the gel based on their biochemical properties. Spots of interest can then be subjected to in-gel digestion for further protein identification. Two of such proteins were identified as participating in energetic metabolism pathways, namely isocitrate dehydrogenase (IDH) and the glycolytic enzyme triosephosphate isomerase (TIM). Additionally, the authors demonstrated upregulated activity by these enzymes in amastigotes when compared to promastigote forms [18].

Isocitrate dehydrogenase is an enzyme that participates in the tricarboxylic acid cycle, a metabolic pathway by which acetate is oxidized to generate ATP. NADP-dependent IDH enzymes catalyze the decarboxylation of isocitrate to α-ketoglutarate, which is accompanied by the production of NADPH. This step is critical in the tricarboxylic acid cycle, and the α-ketoglutarate produced by this enzyme activity can contribute to the synthesis of glutamate, a precursor for amino acids. In this study, the authors showed that IDH-specific activity is approximately three times higher in amastigotes than in promastigotes. As IDH catalyzes the formation of α-ketoglutarate with the production of NADPH, its enhanced activity might be essential for meeting the increased demand for α-ketoglutarate at 37 °C. Triosephosphate isomerase, the other protein exhibiting higher expression in amastigotes, is a highly prevalent enzyme that plays a central role in glycolysis [39]. The authors showed that TIM activity in *L. infantum* amastigotes was two-fold higher compared to *L. infantum* promastigotes, probably because amastigotes require high levels of TIM activity to generate ATP via glycolysis within host cells [18].

In another study, a total of approximately 2000 protein spots were identified in the *L. donovani* proteome, 31 of which were exclusively present in promastigotes [19]. They found 65 proteins with increased expression resulting from heat-induced in vitro amastigote differentiation; however, four proteins exhibited decreased expression in amastigote differentiation. Further studies involving matrix-assisted laser desorption/ionization (MALDI)-time of flight (TOF) and peptide mass fingerprinting revealed 67 protein spots representing 41 different proteins previously identified by databases, in addition to eight hypothetical proteins. In this study, the authors showed that most of the stage-specific proteins identified in *L. donovani* promastigotes or axenic amastigotes can be divided into five groups of proteins with similar function: “(i) stress response (e.g., heat, oxidative stress); (ii) cytoskeleton and cell membrane; (iii) energy metabolism and phosphorylation; (iv) cell cycle and proliferation; and (v) amino acid metabolism” [19]. Although they found interesting data on protein modulation in amastigotes, the authors have yet to validate these data.

Another study with the goal to better evaluate the differentiation of *Leishmania* parasites applied a comprehensive approach consisting of protein prefractionation, followed by global proteomics and targeted DNA microarray analysis. Using 2-D gels, the authors showed that over 2200 protein isoforms were identified in each developmental stage, corresponding to 10% more than what was previously identified by proteomic studies evaluating the in vitro differentiation process of *Leishmania* parasites. Of these, 6.1% were strongly increased or appeared exclusively in the promastigote stage, while 12.4% appeared in amastigotes. Although modest correlations between amastigote-specific protein isoform and mRNA expression (53%) were observed, these authors found no correlation with respect to promastigote-specific spots. They suggested that post-transcriptional controls at translational and post-translational levels may be critically involved in the *Leishmania* parasite differentiation process [40].

As shown by different studies [19,40,41], the major class of proteins exclusively identified or overexpressed in amastigotes was of those involved in stress response or protein folding. Metabolic enzymes were also frequently identified with higher levels of expression in axenic amastigotes compared to promastigotes. In addition, proteins involved in the proteolysis process were also modulated in the amastigote forms [19].

In the first proteomic study performed to evaluate in vitro differentiation of *L. panamensis*, the authors detected 75 differentially regulated protein spots in amastigotes, comprising 24 spots “uniquely” expressed during this life-stage, and 51 that were approximately one to five times overexpressed in comparison to promastigotes [42]. The spots were analyzed by mass spectrometry, and among 11 amastigote-specific spots, six spots were identified as seven distinct proteins. These proteins participate in different cellular processes such as carbohydrate/energy metabolism, stress response, cell membrane and cytoskeleton, amino acid metabolism and cell-cycle. Four additional over-expressed spots were identified as heat shock proteins (HSPs) 60 and 70, and HSP 70-related proteins [42]. Comparative proteomic studies have already shown that proteins involved in stress response and metabolic pathways are differentially expressed among promastigotes and amastigotes from *L. donovani* [19] and *L. infantum* [18].

In a more recent report, a different proteomic approach was performed to study the differentiation process of *L. infantum*. The authors applied protein fractionation by isoelectric point (p*I*) using free-flow electrophoresis to evaluate the expression of stage-specific proteins in this parasite. They identified 2469 protein spots in both life stages. This fractionation process allowed the identification and characterization of several proteins for the first time by proteomic analysis. Glycolytic enzymes and proteins expressed in the parasite flagellum were identified as upregulated in *L. infantum* promastigotes. On the other hand, enzymes involved in gluconeogenesis and fatty acid β-oxidation were upregulated in amastigotes [43]. Additionally, the authors also demonstrated that several proteins were identified in multiple spots, or as proteolytic fragments in both life stages, suggesting the occurrence of post-translational modification and processing.

The first study using a modern quantitative proteomic approach to investigate the differentiation of *Leishmania* parasites, the isotope-coded affinity tag (ICAT) technology associated to mass spectrometry, aimed to identify differentially expressed proteins in *L. infantum* promastigotes and axenic amastigotes. In this report, the authors identified a relatively small number of total and stage-specific proteins. This limited number of proteins was also reported in other recent studies using *L. infantum* [18,44], *L. donovani* [19], and *L. panamensis* [42]. In this work, 8% of the 91 proteins identified were differentially expressed in amastigotes, 20% in promastigotes and 72% were considered constitutively expressed. Proteins with a higher level of expression in amastigotes included two novel proteins and enzymes involved in cell metabolism, as previously shown [45].

One of the branches of proteomics that have become increasingly popular in the last few years is phosphoproteomics. *Leishmania* parasite differentiation requires the activation of signaling cascades involving protein kinases and their downstream phosphoprotein substrates. These signaling pathways are highly adapted to the specific nutritional and physiological requirements of the cells. Therefore, the study of *Leishmania* phosphorylated proteins provided important insights into the parasite biology. Based on these findings, the authors sought to use a gel-based approach in a new study to investigate both qualitative and quantitative changes within the phosphoproteome during the *L. donovani* life cycle stages during in vitro differentiation process [20]. In this pioneering study, phosphoproteins were purified from parasites using immobilized metal affinity chromatography and then separated by 2-DE utilizing fluorescent multiplex staining. The identification of proteins was performed using matrix-assisted laser desorption/ionization-mass spectrometry (MALDI-MS) and mass spectrometry/mass spectrometry MS/MS [20], which identified proteins involved in stress and heat shock response, RNA/protein turnover, metabolism, and signaling. The identification of these proteins reinforces the idea already shown in previous studies that the modulation of proteins involved in stress response and metabolism is critical for *Leishmania* differentiation.

### 2.2. Modulation of Proteins during Intracellular Differentiation of Leishmania Parasites

Until now, the majority of the studies have used axenic parasites grown under in vitro conditions that mimic the sand fly gut (26 °C, pH 7) and phagolysosome (37 °C, pH 5.5) environments to evaluate protein expression in the amastigote stage of the parasite. Although axenic amastigotes are morphologically similar to intracellular amastigotes [46,47,48,49,50,51], a constant concern has been the degree to which axenic amastigotes resemble intracellular ones. Recently, a group performed a comparative proteomic study that evaluated global protein expression in different life stages of *Leishmania*, using amastigotes that underwent the differentiation process from promastigotes to amastigotes intracellularly [52]. The authors used transgenic fluorescent *L. mexicana* parasites that were purified from infected cells combining isopycnic density centrifugation and fluorescent parasite sorting. In this study, a total of 509 different proteins were identified by mass spectrometry, of which 301 were exclusively detected in promastigotes, 31 were only identified in intracellular amastigotes, and 157 were common in both stages. Intracellular amastigotes demonstrated a greater profusion of enzymes involved in the catabolism of fatty acids, which may be the result of this parasite dwelling in acidic compartments, as well as its metabolic adaptation to scarce nutrient availability. These results corroborate those reported for the proteomic analysis of *L. donovani* in axenic amastigotes [53]. In addition, another study that investigated genes, whose products were expressed with higher levels in amastigotes, showed characteristic sequence motifs in 3′-untranslated regions that have been linked to translational control elements, suggesting that proteome data sets may be used to identify regulatory elements in mRNAs. These data support the notion that post-transcriptional processes are important for gene regulation in *Leishmania* parasites [11,12,13,14,17,54].

In a more recent report, an isobaric tagging method was used to quantify the differences among the proteome of promastigotes and amastigotes, which underwent differentiation within human monocyte-derived macrophages (THP-1). The proteins identified as differentially expressed between amastigotes and promastigotes are known to be involved in nutrient acquisition and energy metabolism, cell motility and cytoskeleton, transport, cell signaling and stress response. Upon investigating the proteins involved in vesicular trafficking and endocytosis, such as the rab7 GTP binding protein, GTP-binding proteins of the Ras superfamily, and developmentally regulated GTP-binding protein 1, the authors found enhanced expression in addition to a putative dynein heavy chain protein that was upregulated in amastigotes, which likely plays a role in cargo transport within vesicles. Furthermore, a protein involved in glucose transport exhibited significantly increased expression (8× to 15× higher) in intracellular amastigotes, while several proteins associated with cell motility and cytoskeleton had reduced levels [21].

Taken together, the studies published so far indicate a modulation of the parasite metabolism and molecules involved in stress response after the differentiation of promastigotes to amastigotes, probably favoring the intracellular survival of *Leishmania*.

## 3. Protein Expression by Macrophages in Response to *Leishmania* Infection in Vitro

Only very few studies used proteomics to identify proteins expressed by the host cell in response to *Leishmania*. Tandem liquid chromatography-mass spectrometry (LC-MS/MS) was used to identify markers of resistance and susceptibility in macrophages during *Leishmania* infection in vitro. The CBA mouse macrophages have proven to be useful in identifying these markers because at early time points of infection, the cells present similar percentages of *L. major*- and *L. amazonensis*-infected macrophages. At later time points, a greater proportion of macrophages from the same strain became infected with *L. amazonensis* in comparison to *L. major* [22,55]. A total of 1352 proteins were found expressed in both infected and uninfected CBA macrophages, and only 62 proteins were predominantly expressed in infected macrophages. These proteins were previously described as involved in cell metabolism, or as carrier proteins, in addition to others that participate in cell signaling and cellular detoxification. Another group of proteins contributes to cell immune response, including immune receptors, scavenger receptor class B, and TNF receptor-associated protein. Interesting, only 10 out of the 62 proteins were exclusively identified in *L. major* infection: ribosomal protein S13; glutamate receptor ionotropic; guanine nucleotide binding protein (G protein, γ8 subunit); myosin; proteasome β3 subunit; ras homolog gene family, member B; cytochrome c-1; *N*-acetylglucosamine kinase; TNF receptor-associated protein 1 (TRAP1); and translin. By contrast, the unique protein found expressed in *L. amazonensis* infection was the succinate dehydrogenase, an enzyme involved in cell metabolism. The number of proteins identified in both *L. major*- and *L. amazonensis*-infected cells but that display differences in expression level was much higher, reaching a total of 162 proteins. A total number of 122 proteins were preferentially identified in *L. major*-infected macrophages while only forty of them showed higher expression in *L. amazonensis* infection. When the authors analyzed the greater differences in expression between these infected macrophages, they found a total of 15, 13 of which exhibited reduced expression in response to *L. amazonensis* infection, while two proteins showed increased expression in response to *L. amazonensis* infection.

Considering the 15 proteins with significant levels of differential expression, 13 of these were found downregulated in *L. amazonensis*-infected macrophages, but these were upregulated in *L. major-*infected cells, and they were considered to be involved in several cell processes: coronin 1B, cytochrome C oxidase 6B (cox6B), heterogeneous nuclear ribonucleoprotein F (HNRPF), hypoxia-inducible factor 1-alpha (HIF-1α), osteoclast-stimulating factor-1 (OSTF1), programmed cell death protein 5 (PDCD5), protein phosphatase 2 (PP2), PYD And CARD domain-containing protein (PYCARD), RAB1, ribosomal protein S2 (RPS2), Serpin, peripheral benzodiazepine receptor (PBR), known as translocator protein (TSPO), and myosin light chain. The authors organized the identified proteins in networks using a biological network modeling, the Ingenuity Pathway Analysis (IPA)-Ingenuity Systems. This tool allows the organization of proteins detected in proteomic studies, as well as other proteins that are not identified by the mass spectrometric analysis, but may be involved in host response to infection. Interestingly, 14 out of 15 proteins with significant levels of differential expression were organized into a single network of cell development.

Between the two highly expressed proteins in CBA macrophages infected with *L. amazonensis*, one of them, phospholipase D1 (PLD1), was proven to act on the membrane phospholipid, phosphatidylcholine. This protein causes the release of phosphatidic acid [56], as well as participates in the recruitment of additional membrane for the formation of nascent phagosomes. This protein can take part in the maintenance of early formed phagosome that will fuse with endocytic vesicles [57]. The authors suggested that the higher expression of PLD1 in *L. amazonensis*, but not in *L. major* infected macrophages, would contribute to the formation and maintenance of large parasitophorous vacuoles, characteristic of intracellular infection with *L. amazonensis* [58].

In this study [58], two out of the 15 proteins with a higher difference of expression were randomly selected for the validation of mass spectrometry results. Myosin light chain was validated as highly expressed in *L. major*-infected cells compared to *L. amazonensis*-infected macrophages using western blot and immunofluorescence staining for HIF-1α, which confirmed a higher expression of this protein in *L. major*-infected cells compared to those infected with *L. amazonensis*.

The finding that myosin light chain was upregulated in *L. major*-infected macrophages was related to the formation of small individualized parasitophorous vacuoles induced and maintained throughout the maturation process in *L. major* infection [58], different from the large parasitophorous vacuoles that *L. amazonensis* induces in host cells. Previously, this protein has been implicated in the formation and maintenance of tight vacuoles formed around particles during phagocytosis [59].

Modulation of immune response was evidenced by HIF-1α, TRAP1, Serpin and PYDCARD that were upregulated in *Leishmania*-infected macrophages. TRAP1 and HIF-1α were found highly expressed in macrophages infected with *L. major*, and Serpin and PYDCARD exhibited reduced expression levels under *L. amazonensis* infection. TRAP1 has been shown to participate in the maintenance of cellular viability in cells subjected to H_2_O_2_-induced oxidative stress [60], and Serpin, a protein induced by TNF-α known to participate in conjunction with IL-1α in the inflammatory cascade [61]. Additionally, the PYDCARD adapter protein activates apoptosis by a mechanism dependent on NF-κB and caspases [62] that is initially induced by engagement of receptors in the TNF-α family. These proteomic findings can also be correlated with a previous study that CBA macrophages, which control *L. major* infection, express twice as much TNF-α when infected with *L. major* compared to those infected with *L. amazonensis* in response to IFN-γ stimulation [63]. Finally, the finding that HIF-1α is highly expressed in *L. major*-infected macrophages suggests a relationship between this transcription factor expression and a higher production of NO and expression of TNF-α, which are mediators known to act as regulators of HIF-1α when expressed under normoxic conditions [64]. The roles that myosin light chain and HIF-1α play in *Leishmania* infection deserve further investigation.

Another study that evaluated the host cell response to *Leishmania* infection using proteomics was recently published. The study, which was a quantitative proteomic study, aimed to evaluate the effect of *L. donovani* parasites on the host cell response [23]. In this study, the authors infected the THP-1 with *L. donovani* promastigotes and following infection they determined relative and absolute quantification of protein expression using the isobaric tags (iTRAQ) method and LC-MS/MS. They compared the expression profiles of non-infected and *L. donovani*-infected THP-1 cells and found that proteins involved in major metabolic pathways, including glycolysis and fatty acid oxidation, are highly expressed after *L. donovani* infection, suggesting a parasite-induced global reprogramming of cell metabolism. The expression of proteins involved in gene transcription, RNA splicing (heterogeneous nuclear ribonucleoproteins (hnRNPs)), histones, and DNA repair and replication was also found to be increased in response to *L. donovani* infection in vitro. Proteins involved in cell survival and signal transduction were also shown to be more abundant in response to infection. Interestingly, several of the proteins that were identified as differentially expressed in this study had not been previously associated with the host response to the parasite, while some were related to proteins identified in previous studies. Quantitative polymerase chain reaction and immunoblot analysis of selected proteins identified in the study were used to validate proteins found differentially expressed in the mass spectrometry study. RNA interference (RNAi)-mediated gene knockdown of proteins was used to confirm the relevance of the observed host quantitative proteomic screening.

Interestingly, this study shows that the mitochondrial antiviral signaling protein (MAVS) was significantly abundant in host cells after *Leishmania* infection. MAVS, the first mitochondrial protein to activate NF-κB and interferon (IFN) regulatory factors (IRF3 and IRF7), is known to synthesize type I interferons (IFN-α and IFN-β), which are essential in antiviral signaling. The silencing of endogenous MAVS expression by RNAi inhibits the activation of NF-κB, IRF3, and IRF7, thereby blocking the production of interferon and promoting viral infection [65]. These authors claim that there may be cross-talk between MAVS and the components of the NF-κB and IRF signaling pathways, which leads to the production of proinflammatory cytokines and type I IFN [66].

A recent study published using phosphoproteomics showed that *Leishmania* parasites respond to arginine pool reduction in the host cell by up-regulating expression and activity of the Leishmania arginine transporter (LdAAP3), as well as several other transporters. To study phosphoproteins involved in the signaling pathway that results in increased LdAAP3 expression and activity, the authors used a di-methylation tagging technique to evaluate changes in the phosphorylation profile of *Leishmania* promastigotes after five and 15 min of arginine deprivation. Phosphorylation analysis showed an increased phosphorylation of mitogen-activated protein kinase 2 (MPK2), suggesting that the arginine-deprivation response during *Leishmania* infection is mediated through a MPK2-dependent signaling cascade [67].

Taken together, these three studies show that *Leishmania* parasites modulate the host cell proteome during early stages of infection, providing evidence for the complex relations between the host and the parasite at the molecular level. These studies also reveal potential novel cellular targets which could modulate cell response to help control *Leishmania* infection.

## 4. Protein Profiling in Human Cutaneous Lesions

A recent study generated interesting results in tegumentary leishmaniasis using 2-DE proteomics, as well as biological network analysis for protein profiling in cutaneous lesions comparing protein expression to normal skin samples [26].

In this study, the authors found that cutaneous lesions showed a composition similar to the inflammatory infiltrate with the presence of lymphocytes, macrophages, and plasma cells, as well as focal necrosis. Proteins that were extracted from lesions and normal skin samples were separated using 2-DE. Among a total of 150 differentially expressed spots of proteins from cutaneous lesions and normal skin samples, the authors identified 59 proteins. From these, 29 spots were identified only in CL samples, while 17 were found only in the normal skin. Besides, among the spots present in both cutaneous lesions and control samples, nine spots were upregulated, and four were downregulated in CL biopsies in comparison to normal skin.

The proteins identified in the mass spectrometry analysis were organized according to biological functions, and the analysis revealed that those upregulated in CL biopsies participate in several cell processes including apoptosis (caspase-9 (CASP-9)), immune response (T cell receptor beta (TRB)), and biosynthetic process (transcription factor IIIB 90 kDa subunit (BRF1)). Previously, it was observed that an increased expression of TRB [68,69] could be involved in the recruitment of T cells to the infection site. The authors claimed that the increase in the apoptotic process, consequent to CASP-9 expression could be the result of the development of an intense inflammatory response commonly observed in cutaneous lesions of leishmaniasis.

In this work, the authors performed biological network analysis to better understand how biological pathways were modulated in infected individuals. This approach was also used in the in vitro study using mouse macrophages [22]. To perform this analysis, the authors generated networks of interactions between the identified proteins, in addition to other proteins, which were included in the network by the Cytoscape software. A complex network was generated by Cytoscape, containing 505 nodes that contained many proteins involved in apoptosis, such as IL-23, transforming growth factor beta receptor 1 (TGFBR1), tumor necrosis factor receptor-I (TNFRI), caspase-3 (CASP-3), caspase-8 (CASP-8), and granzyme B (GZMB).

Reinforcing the role that the apoptotic process plays in CL development, IPA generated the cytotoxic T lymphocyte-mediated apoptosis of target cells as the primary canonical pathway. This pathway included 34 proteins, 18 of which were differentially expressed among the samples. Nine out of these 18 proteins were exclusively expressed in CL biopsies, and five were upregulated, while four were downregulated, in CL biopsies when compared to normal skin.

In the apoptosis cascade, granzyme B is a protein that has been shown to activate caspase-3, either directly or via the mitochondrial pathway, by way of induction of caspase-9 activation, which subsequently activates caspase-3 [70]. These authors validated the expression of three proteins, two of which were identified as differentially expressed in their proteomic study and another that was identified by IPA. Using immunohistochemistry, they demonstrated higher expression of caspase-9, caspase-3, and granzyme B in CL biopsies in comparison to normal skin. Also, they found that higher expression of these three proteins correlated positively with lesion size in CL patients. They conclude that apoptosis is probably involved in the mechanisms associated with the progression of tissue damage seen in CL lesions.

## 5. Protein Expression in Serum of Individuals with Visceral Leishmaniasis 

Very few studies have used large-scale analysis to better understand the aspects surrounding host immune inflammatory response to *Leishmania* infection. Recently, three studies have used proteomics to identify biomarkers that potentially participate in host immune response in the serum of VL patients [28,29], in addition to those that may help in the diagnosis of visceral disease [27]. 

Two proteomic studies employing either a quantitative [28] or a qualitative comparative [29] analysis identified differentially expressed proteins in human serum from patients and control groups. The patient study groups were diagnosed with VL as confirmed by the presence of *Leishmania* parasites in bone marrow aspirate [28,29] or by the presence of serum anti-rK39 antibody [28,29]. 

The first study using a quantitative proteomic approach evaluated the proteome in the serum of six VL patients and six healthy volunteers. The authors identified 26 differentially expressed spots, from these 15 were analyzed by mass spectrometry and only nine spots were identified with high confidence, corresponding to five different proteins. In the comparative proteomic study, the authors purified only plasma glycoproteins using a multi-lectin affinity column, followed by mass spectrometry analysis that allowed for the identification of 39 differentially expressed spots [29].

In both quantitative [28] and qualitative comparative [29] studies, some acute-phase proteins were found to be differentially expressed in sera from humans with VL compared to controls, supporting the notion that VL is a systemic disease. The glycoproteins identified as upregulated in only the qualitative comparative proteomic study were α-1-acid glycoprotein and C1 inhibitor, while, in the quantitative study, α-1-antitrypsin, α-1-B glycoprotein, and amyloid-A1 precursor were detected. On the other hand, the only downregulated protein identified in the qualitative glycoproteomic study, was the retinol binding protein, while, in the quantitative study, the vitamin-D binding protein was detected. Interestingly, in both studies, apolipoprotein A-I and transthyretin were found to be downregulated in VL sera [28,29].

Previously, α-1-acidglycoprotein was found to be elevated in sera of individuals with systemic tissue injury, or during inflammation and infection. This protein was also proven to be involved in other processes, such as neutrophil inactivation, chemotaxis and oxidative metabolism [71]. The authors claimed that the increased levels of the acute phase α-1-acid glycoprotein observed in serum from VL patients can result in the inhibition of neutrophil function, facilitating pyogenic infections in the skin and other tissues [28]. C1-inhibitor was the other acute-phase protein found to be overexpressed in serum from VL patients [28]. Inhibition of both complement and quinin generating cascades [72] by this plasma protease inhibitor has been associated with the anti-inflammatory effect of C1-inhibitor [73].

One of the proteins found to be downregulated in sera from VL patients was transthyretin [28], which is known to function as a transporter of thyroid hormones, as well as a negative acute phase protein with anti-inflammatory properties that subsequently results in inhibition of interleukin-1 production by monocytes and endothelial cells [74]. In agreement with this proteomic study, a previous one found transthyretin levels reduced in sera of patients with VL [75]. The other protein found downmodulated in the sera from VL patients was the retinol binding protein [28], known to transport retinol from the liver to the peripheral tissues. In plasma, retinol binding protein interacts with transthyretin, preventing retinol binding protein loss through filtration in the kidney [76].

Another proteomic study was performed to identify proteins potentially expressed by parasites in dogs with VL, as well as differences in protein expression in serum from infected and non-infected dogs [27]. The study performed in naturally infected dogs demonstrated that the mass spectrometry was not sensitive enough to detect parasite protein in dog sera. Nonetheless, labeling each sample of infected and non-infected individuals with different iTRAQ tag followed by the LC-MS/MS analysis allowed the authors to identify differentially expressed host proteins in serum of infected animals when compared to the non-infected ones. More than 105 proteins were identified in the serum of dogs, of which 22 were found in sera from infected dogs compared to controls. Of these 22 proteins, 17 were upregulated, and five were downregulated [27].

The number of proteins identified in the serum of dogs in this proteomic study [27] was greater than the number found in the proteomic studies using human serum [28,29]. Despite differences in the number of proteins expressed in dogs and humans with VL, increased levels of acute-phase proteins were identified as modulated in the three studies [28,29]. The proteins found in dog serum with VL, such as serum amyloid A (SAA) and haptoglobin, were different from those detected in human serum. The modulation of acute-phase proteins is consistent with results from previous studies, both in canine [77] and in human VL [78]. Furthermore, in patients with VL, the increased levels of such acute phase proteins in serum progressively decreased with effective therapy, while the high expression levels were maintained in those patients with parasite clearance delay [78].

In these proteomic studies, the finding that the inhibition of negative acute-phase proteins along with the enhancement of positive acute-phase protein expression in sera from humans and dogs with VL reinforces the notion that infection leads to an uncontrolled systemic inflammatory response. This response can account for the pathogenesis and clinical manifestations of VL [79]. The different studies postulate that the identification of these molecules could be useful as diagnostic/prognostic biomarkers and in the understanding of parasite survival in the host environment [29]. Additionally, these molecules could function as potential targets for future development of new treatment for controlling the clinical manifestations of the disease [28]. Finally, the study that found a reduction in the expression of acute-phase proteins in response to antileishmanial therapy [77] reinforces the potential use of these molecules as targets for monitoring the initial response to treatment and follow-up of humans and dogs with VL.

## 6. Concluding Remarks

**Figure 3:**
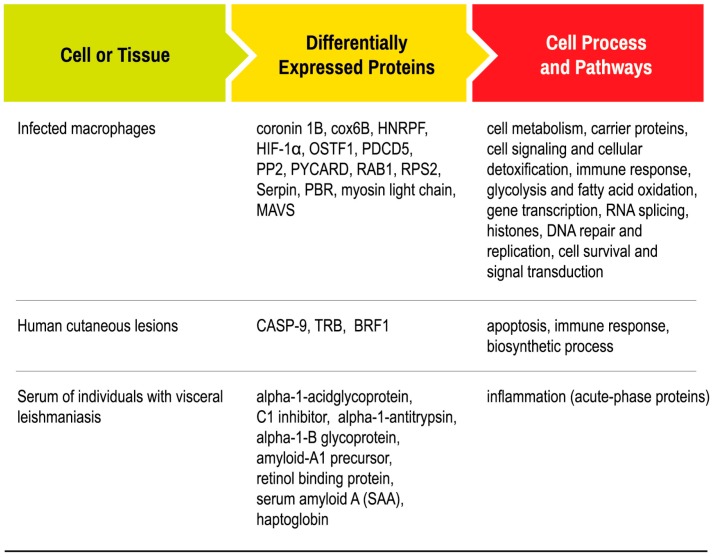
Proteins involved in the host response to *Leishmania* infection. Proteins identified as differently expressed in *Leishmania* infected host in proteomics studies.

Comparative proteomics studies have demonstrated that the relation between *Leishmania* parasites and the host is extremely complex at the molecular level. Although very few proteins have been identified exclusively in *Leishmania* amastigotes, a consistent number of proteins modulated in this life stage were involved in metabolic pathways. In addition, during the host-parasite interaction, the expression of proteins in the host varies depending on the *Leishmania* spp., as well as the tissue or cell studied (Figure 3). However, most studies reported that the proteins involved in metabolic pathways and the immune and inflammatory response of the host are frequently modulated.

To this date, none of the molecular targets identified in proteomic studies were used to develop new treatment approaches or as prognostic markers in a follow-up of different types of *Leishmania* infection. This review will provide a reference to conduct new studies to better understand the role these proteins and pathways play in the intracellular survival of *Leishmania* parasites and the outcome of leishmaniasis.
